# A Bayesian Supertree Model for Genome-Wide Species Tree Reconstruction

**DOI:** 10.1093/sysbio/syu082

**Published:** 2014-10-03

**Authors:** Leonardo De Oliveira Martins, Diego Mallo, David Posada

**Affiliations:** Department of Biochemistry, Genetics and Immunology, University of Vigo, Vigo, 36310, Spain

**Keywords:** hierarchical Bayesian model, phylogenomics, reconciliation, supertree, tree distance

## Abstract

Current phylogenomic data sets highlight the need for species tree methods able to deal with several sources of gene tree/species tree incongruence. At the same time, we need to make most use of all available data. Most species tree methods deal with single processes of phylogenetic discordance, namely, gene duplication and loss, incomplete lineage sorting (ILS) or horizontal gene transfer. In this manuscript, we address the problem of species tree inference from multilocus, genome-wide data sets regardless of the presence of gene duplication and loss and ILS therefore without the need to identify orthologs or to use a single individual per species. We do this by extending the idea of Maximum Likelihood (ML) supertrees to a hierarchical Bayesian model where several sources of gene tree/species tree disagreement can be accounted for in a modular manner. We implemented this model in a computer program called *guenomu* whose inputs are posterior distributions of unrooted gene tree topologies for multiple gene families, and whose output is the posterior distribution of rooted species tree topologies. We conducted extensive simulations to evaluate the performance of our approach in comparison with other species tree approaches able to deal with more than one leaf from the same species. Our method ranked best under simulated data sets, in spite of ignoring branch lengths, and performed well on empirical data, as well as being fast enough to analyze relatively large data sets. Our Bayesian supertree method was also very successful in obtaining better estimates of gene trees, by reducing the uncertainty in their distributions. In addition, our results show that under complex simulation scenarios, gene tree parsimony is also a competitive approach once we consider its speed, in contrast to more sophisticated models.

The evolutionary history of a gene family is not an exact representation of the evolution of the species embedding it, due to the effects of gene duplication, deep coalescences—also called incomplete lineage sorting (ILS)—and horizontal gene transfer (HGT) (Goodman et al. 1979; Maddison 1997). In recent years, the rapid accumulation of phylogenomic data has highlighted the need for specific methods to infer species phylogenies. Methods of species tree inference based on collection of gene alignments/trees can be broadly classified into supermatrix, supertree, and model-based approaches. The supermatrix strategy consists in concatenating different genes into a single large alignment, assuming that minor conflicting phylogenetic signals will cancel out and a common phylogenetic trend—the species history—will emerge. Departures from this assumption must be handled by subjectively removing columns or whole regions (Wu et al. 2012; Lanier et al. 2014). The supermatrix approach relies on the correct inference of the orthologous sets, such that the sequences from a single species can be concatenated into a single ‘supergene’ (Degiorgio and Degnan 2010).

Alternatively, phylogenetic inference can be conducted independently for each gene alignment, and then in a second step the species tree is inferred from the resulting gene trees. In this case, the alignments are used indirectly, through summary statistics like ML trees, to build a ‘supertree’ (for a review, see Bininda-Emonds et al. 2002; Cotton and Wilkinson 2009). Under the supertree umbrella, some methods try to find the species tree that minimizes its overall disagreement with the gene tree collection without explicitly—taking into account the biological phenomena behind the incongruence, like the Robinson-Foulds (RF) supertree (Bansal et al. 2010; Chaudhary et al. 2012) and the Matrix Representation with Parsimony approaches (Ragan 1992; Bininda-Emonds 2004). Other supertree approaches aim for the most parsimonious scenario by assuming that the disagreement between the species tree and the gene trees is due to HGT (Whidden et al. 2014), duplications and losses (DL) or deep coalescences (Bansal and Eulenstein 2013). The problem of finding the species tree that minimizes the reconciliation cost is called gene tree parsimony (GTP) (Guigó et al. 1996).

Most supertree approaches neglect the gene tree branch lengths, but there is another class of species tree methods closely related to the supertree methods which are based on the multispecies coalescent. These methods try to reconstruct the species tree from a matrix of distances between species, which in turn is built based on distance matrices from the individual gene trees (reviewed in Liu et al. 2009a; Helmkamp et al. 2012). These methods are based on the observation that the coalescence time of two genes from distinct species always precedes the speciation event. In general they assume that the gene trees are known without error (Kubatko et al. 2009; Liu and Yu 2010). Some of these methods also can take into account hybridization (Meng and Kubatko 2009) or HGT (Yu et al. 2011).

In principle, the most powerful species tree methods are based on an explicit probabilistic modeling of the incongruence between species and gene trees. Together with the phylogenetic likelihood that takes into account the stochastic uncertainty in the gene tree inference, these models also describe the probability of such gene tree being generated by a given species tree. If we assume that the sequences represent individuals from a given orthologous region, then this probability is given by the multispecies coalescent, that describes how lineages coalesce conditioned on a species tree (Rannala and Yang 2003). Under this model, Bayesian methods have already been implemented to estimate the posterior distribution of species trees, together with ancestral population sizes and divergence times (Edwards et al. 2007; Heled and Drummond 2010). If, however, we assume that each alignment represents a set of paralogous gene copies from a gene family, then we can model DL inside each gene family through a birth–death process (Arvestad et al. 2003). Although theoretically it is possible to estimate the distribution of species trees while integrating over the particular birth–death histories, current implementations assume that the species tree is known (Akerborg et al. 2009; Rasmussen and Kellis 2011; Sjostrand et al. 2012) or try to find it by ML (Boussau et al. 2013). Under the fixed tree assumption, it is furthermore possible to add HGT to the birth–death model (Szollosi et al. 2013). Fully probabilistic models, although more realistic, tend to be slow. Furthermore, they are usually limited to particular sources of gene tree / species tree disagreement. Under the multispecies coalescent, it is assumed that all genes from same species are orthologous, neglecting the possibility of duplication, whereas duplication and loss models assume that sequences mapped to one species are necessarily the product of a duplication (Rasmussen and Kellis 2012).

Recently a ML supertree approach has been proposed, such that probabilities are associated to errors in the gene trees (Steel and Rodrigo 2008; Cotton and Wilkinson 2009). That is, given a supertree the probability will reflect a penalty against incongruent input trees, regardless of the evolutionary process responsible for the incongruence. The model assumes that a true supertree generates trees with error, such that their probabilities decrease exponentially according to the distance from the original supertree. The incorporation of supertree approaches into a likelihood framework allows us to compare alternative solutions statistically and to be more explicit about our assumptions, incorporating model selection and hypothesis testing. The probability $P(T_{i}|\tau ,\lambda )$ of supertree τ generating tree $T_{i}$ is given by

(1) $$P(T_{i}|\tau ,\lambda )\propto e^{-d(T_{i},\tau )/\lambda }$$

where the scale parameter λ controls how strongly discordant trees are penalized. The distance d() is usually a discrete tree-to-tree metric that does not take branch lengths into account, and therefore the original ML supertree approach is based on tree topologies only. Furthermore, it is assumed that each input tree contains a subset of the species, as is commonplace for supertree methods. The particular distance employed will depend on the problem at hand, as for example, the RF distance will lead to a supertree in agreement with the largest number of clusters (or clades) from the input trees (Bansal et al. 2010). Likewise, the subtree prune-regraft (SPR) distance (Beiko and Hamilton 2006) has been used in an exponential model of phylogenetic recombination, where the penalty is against trees in adjacent alignment segments that can only be reconciled through several recombination events, as estimated by the SPR distance between them (de Oliveira Martins et al. 2008; de Oliveira Martins and Kishino 2010).

Probabilistic supertree approaches like ML supertrees have a lot of potential, as they can leverage statistical sophistication with computational tractability, being able to deal with several processes of incongruence at once (Cotton and Wilkinson 2009). In this work, we further extended the ML supertree approach in several notable ways, creating a hierarchical Bayesian supertree model. We notice that an estimation method called “Bayesian supertree” was proposed by Ronquist et al. (2004), but it is quite different from our model since it is based on frequencies of bipartitions only. First, we use a multivariate description of the disagreement between each input tree and the supertree, such that several distances can be used at once. Importantly, among the distances we use jointly are the most parsimonious reconciliation costs under the DL and the ILS models (Guigó et al. 1996; Than and Nakhleh 2009. This multivariate distribution acts as a penalty against dissimilar input tree/supertree pairs according to any of the distances, such that input gene trees less penalized with respect to all distances from a given supertree are more likely.

In our model, the input trees represent gene families and the supertree is the species phylogeny. This allows us to work with more general data sets than traditional supertree methods usually handle, since we can work with input trees with several leaves representing the same species (in fact, input trees can have more leaves than the supertree itself), as well as nonoverlapping species subsets for distinct gene families. Furthermore, we incorporate this multivariate distribution of tree-supertree distances into a hierarchical Bayesian model, such that the uncertainty of the gene trees as well as the strength of the penalties — represented by the scale parameter λ — are taken into account.

Our full model can include the phylogenetic likelihood describing how gene trees are supported by the alignments, but in this manuscript we adopted an importance sampling step such that any Bayesian phylogenetic program can provide the individual distribution of gene trees. That is why we call our method a supertree, in connection to the ML supertree (Steel and Rodrigo 2008), despite the fact that our full model could in principle work with sequence alignments as well. Our model accounts then for the uncertainty within each gene family to estimate the posterior distribution of all compatible species trees. Unlike the fully probabilistic models described above, our approach is based on the simplifying assumption that summaries based on the most parsimonious reconciliation scenarios are sufficient to explain the disagreement between gene and species trees, regardless of its causes. Yet we do not rely on known gene trees, as with most supertree approaches. Instead, we work with marginal gene tree distributions such as those resulting from independent Bayesian phylogenetic analyses of multilocus alignments. We implemented this model in a program called *guenomu* (see below), that receives as input a set of unrooted gene tree distributions, one per gene family, as well as a list of species names, and returns the posterior distribution of rooted species trees as well as a posterior distribution of gene trees for each gene family.

In this manuscript, we evaluate our method under simulated scenarios in which gene trees evolve inside species tree with gene DL as well as ILS, and considering gene tree uncertainty. Usually, different sources of gene tree disagreement are considered separately, although unrecognized processes can have a detrimental effect on the species tree estimation, since even a single ancestral duplication can mimic the effect of several deep coalescences (Rasmussen and Kellis 2012). We circumvent this by favoring species trees that are compatible with different phenomena at once, as dictated by our multivariate distance distribution. Each gene family is allowed to have different contributions in each regard, since we do not expect the rates of DL, for instance, to be uniform along the genome. Our simulations suggest that our Bayesian hierarchical model is able to reduce gene tree uncertainty and results in accurate species trees estimates, considering the complexity of the simulation scenario. Unexpectedly, we also found that GTP methods can be quite precise as well, whereas coalescent summary statistics methods performed badly under the scenarios considered.

## Materials and Methods

### Definitions

We define *gene family* i as the set of homologous sequences that compose an alignment $D_{i}$, which can comprise paralogs and orthologs belonging to the same or distinct individuals from one or more diverged populations, that we will call *species*. The only information needed is a mapping between each member of this gene family and the species which it belongs to (species are thus defined as the entities whose phylogenies we are interested in). Importantly, we assume that we do not know beforehand which members of a gene family are related through a duplication and which diverged through coalescences. That is, we map the leaves from the gene tree directly to the species tree. We will then call *gene tree* any phylogenetic tree connecting all sampled members of a gene family, whose corresponding random variable for gene family i will be represented by $G_{i}$. The inputs are posterior distributions of gene trees such as those estimated by Bayesian phylogenetic inference programs like MrBayes or PhyloBayes (Lartillot and Philippe 2004; Ronquist et al. 2012). These will be our *input gene tree distributions*, one per gene family, and for each input distribution we will have an associated *posterior distribution*, in a manner analogous to the BUCKy model (Ané et al. 2007). Although we will mostly use the term *tree*, the input and output distributions contain only the tree topologies. That is, in the current implementation of our model we do not use the branch length information, unless otherwise stated. We will resort to the terms *phylogeny* and *topology* when we want to be explicit about the presence or absence, respectively, of branch lengths.

### Bayesian Hierarchical Model for Species Trees

To gain some intuition about our model, we start by observing that a Bayesian model is comprised of the product between the *likelihood* (the probability terms containing the data D) and the *prior* for the parameters, divided by the (marginal) probability of the data. The latter usually cannot be computed analytically, and one typically resorts to Markov chain Monte Carlo (MCMC) algorithms to avoid its calculation. Whenever the data can be partitioned into independent components, each partition is included as a likelihood term in the posterior distribution. The parameters from each likelihood term can be shared across components or be specific to a partition. When the parameters are the same for all partitions it is enough to describe their shared prior, that will depend on a single *hyperparameter* (which can be multidimensional). But usually it is more reasonable to assume that each partition has its own set of parameters and therefore its own prior. These priors can, again, have independent hyperparameters of their own, or share them at least partially with other partitions. A hierarchical Bayesian model is one where other layers of priors are added to control how the different parameters are related, assuming that parameters from one partition can inform the others through their common underlying structure. The latter is done through the incorporation of *hyperpriors*, that describe the distribution of the hyperparameters and which, in turn, might depend on so-called *hyperhyperparameters*—parameters from the hyperprior—that can be fixed or follow a *hyperhyperprior*, and so forth. The hierarchical model is completed once it can be assumed that adding more layers will not affect the inference of parameters. Each parameter can in fact be a matrix of arbitrary dimensions, and each component can be partitioned in its own way, according to what information is believed to be shared across partitions and what information is particular to a given partition. An excellent description of hierarchical Bayesian models in phylogenetics is given by Suchard et al. (2003).

In our model, the phylogenetic likelihood is given by the probability of the alignment D given the phylogenetic tree G and the substitution parameters, as usual (Felsenstein 1981). The priors will describe the probability distributions for the substitution parameters (base frequencies, transition rates, rate variation across sites, etc.) and for the phylogenetic tree. Assuming that the alignment represents a gene family, its corresponding gene tree will represent the evolution of its members—ortholog/paralog gene copies from different individuals and/or loci, assumed to share a common ancestry. We consider that every gene tree is embedded in a species tree, so the prior probability for each possible gene tree generating the observed alignment must take into account the evolutionary history of the species represented. This is where the error-based probability of Steel and Rodrigo (2008) enters into our model: it tells that the prior probability of a gene tree is proportional to how it resembles the underlying species tree. We however demand that this resemblance takes into account several biologically sensible measures of disagreement, which can contribute distinctively through different *penalty* parameters. These measures of gene tree/species tree disagreement can be the number of DL or the number of deep coalescences, and the penalty parameters describe how strictly we penalize dissimilar gene/species tree pairs. Note that in our model the species tree becomes then a hyperparameter, that we furthermore assume to come from a fixed uniform hyperprior over all possible species trees with the same number of taxa. The penalty parameters are also hyperparameters, but whose hyperpriors are not fixed. This is because if several gene families are available, then it is natural to partition them since they can obviously have distinct gene trees, as well as their own substitution parameters. However, their prior distributions of gene trees should share the same species tree, even if they have their own penalties. Our intuition tells us that the more gene families we include, the more information about the species tree we can obtain. The same cannot be said about the penalty parameters, because for every gene family we must add a set of parameters. To avoid overparameterization we assume that, for each given gene tree/species tree disagreement measure, the penalties from all gene families come from a common hyperprior, that represents the genome-wide effect of the biological phenomenon causing the gene tree/species tree disagreement. These genome-wide associated hyperhyperparameters, however, must be allowed to vary if we want the amount of duplications or deep coalescences inferred from one gene family to be able to inform other gene families through their shared hyperprior.

To summarize our intuition, our model assumes that each gene family (represented by an alignment) was generated by an independent gene tree, but that these trees from different gene families all share the same species tree. Furthermore each gene family tree cannot be too different from the species tree according to several distances, and while the penalties are specific to a gene family, they also share a common prior for each distance.

We have then devised a Bayesian hierarchical model in which the posterior distribution of the species tree S and all other parameters $\Theta =(\theta ,G,\lambda ,\lambda_{0})$ given a set of N gene family alignments $D=(D_{1},...,D_{N})$ can be described by

(2) $$P(S,\Theta |D)\propto P(D,\theta |G)P(G|\lambda ,S)P(\lambda |\lambda_{0})P(\lambda_{0})P(S)$$

where $G=(G_{1},\ldots ,G_{N})$ are the gene trees and $\lambda =(\lambda_{ij})$ is a matrix with penalty parameters j for gene family i, which depend on the (hyperhyper)parameters $\lambda_{0}$. The term P(D,θ|G) is the joint probability of alignments D and parameters θ related to the substitution model, given the gene trees—it is the product of the likelihood and the prior for θ. As we will see this calculation is delegated to the user, so our model works equally for DNA, codon, and protein alignments. All species trees S are equally probable a priori, leading to the uniform prior $P(S)=|S{|}^{-1}$ over all possible rooted trees S∈S. Equation 2 can be further decomposed into

$$P(S,\Theta |D)\propto P(\lambda_{0})P(S)\times \prod_{i=1}^{N}P(D_{i},\theta_{i}|G_{i})P(G_{i}|\lambda_{i\cdot },S)P(\lambda_{i\cdot }|\lambda_{0})$$

where we see that the scale parameters $\lambda_{0}$ and the species tree S are shared among all gene families, whereas other parameters are allowed to vary between families.

Steel and Rodrigo (2008) assumed that the probability $P(G_{i}|S)$ of a gene tree $G_{i}$ given the species tree S followed an exponential distribution that depended solely on the dissimilarity between $G_{i}$ and S, which can be represented by a distance $d(G_{i},S)$ and a penalty parameter $\lambda_{i}$. We generalize this distribution and assume that this probability depends on the disagreement between $G_{i}$ and S with respect to several distances $d(G_{i},S)=(d_{1}(G_{i},S),\ldots ,d_{J}(G_{i},S))$ (explained in detail below) where each measure $d_{j}(j=1,...,J)$ represents a distinct distance. Therefore, the distribution $P(G_{i}|S)$ can be written as

(3) $$P(G_{i}|S)=\frac{e^{-\sum_{j=1}^{J}d_{j}(G_{i},S)/(m_{ij}\lambda_{ij})}}{\sum_{g\in G_{i}}e^{-\sum_{j=1}^{J}d_{j}(g,S)/(m_{ij}\lambda_{ij})}}=\frac{e^{-\sum_{j=1}^{J}d_{j}(G_{i},S)/(m_{ij}\lambda_{ij})}}{Z_{i}(S,\lambda_{i\cdot })}$$

where $Z_{i}(S,\lambda_{i\cdot })$ is the *normalization constant* (also called partition function; see below) over the set $G_{i}$ of all possible topologies for gene family i, and the $m_{ij}$ are fixed constants.

Each gene family i has a vector $\lambda_{i\cdot }=(\lambda_{i1},\ldots ,\lambda_{iJ})$, where each parameter $\lambda_{ij}$ is associated to a distance $d_{j}$. Our model allows for different parameterizations depending on which distances we want to consider for a specific analysis.

Our hierarchical model is completed by specifying the priors for the distance penalty parameters. For each distance j, the penalty parameter $\lambda_{ij}$ of gene family i follows an exponential hyperprior distribution

(4) $$P(\lambda_{ij}|\lambda_{\text{0}})=\frac{e^{-\lambda_{ij}/\lambda_{\text{0}j}}}{\lambda_{\text{0}j}}$$

where the penalty parameter $\lambda_{\text{0}j}$ is the j-th element of the vector $\lambda_{0}$, which is shared among gene families.

As mentioned in Steel and Rodrigo (2008) a naive estimation of the $\lambda_{i\cdot }$ penalty parameters will lead to very permissive values, and thus we employ an informative hierarchical exponential model for them—that is, we explicitly inform the model that we prefer small distances overall. The $\lambda_{\text{0}j}$ variables themselves come from exponential distributions with a fixed parameter. Because distinct gene families will usually have a different number of members, we must ensure that penalty parameters $\lambda_{ij}$ can be comparable across gene families. This is achieved through the scaling parameters $m_{ij}$, which are equivalent to a standardization of all distances $d_{j}(\cdot ,\cdot )$ to the interval [0,1]. The choice of a common prior parameter $\lambda_{\text{0}j}$ shared across gene families helps to avoid overparameterization. But more importantly, it assumes that the underlying biological process reflected by the distance j—like the duplication rate, for instance—acts on the whole genome, whereas at the same time the $\lambda_{ij}$ parameters for the observed distances are particular to each gene family i.

### Measures of Disagreement

In our current implementation, we have explored two groups of distances, which we will call *reconciliation distances* and *nonparametric distances* (although they are not proper metrics because they do not satisfy the symmetry condition). The reconciliation distances are based on the most parsimonious reconciliations between the rooted species tree and gene trees (Page 1994; Maddison 1997), which can be calculated using the last common ancestor (LCA) mapping between each node g of the gene tree and a corresponding node M(g) of the species tree (Zmasek and Eddy 2001; Bansal and Eulenstein 2013). This mapping can thus be used in two independent ways: to find the minimum number of DL needed to explain the disagreement (Guigó et al. 1996; Zhang 1997), or to find the minimum number of deep coalescences necessary to make the gene tree be congruent with the species tree (Than and Nakhleh 2009; Wu and Zhang 2011).

Under the duplication-loss reconciliation model and given the mapping M(·) between nodes of the gene tree $G_{i}$ rooted at r, denoted $G_{i}^{r}$, and the rooted species tree S, a gene tree node g will represent a duplication if $M(g)=M(g_{1})$ or $M(g)=M(g_{2})$, where $g_{1}$ and $g_{2}$ are the two children of node g. The number of duplications $\text{DUPS}(G_{i}^{r},S)$ will be the number of such nodes, and the number of losses $\text{LOSS}(G_{i}^{r},S)$ is calculated in postorder based on the LCA mapping and the duplication nodes. Likewise, under the reconciliation coalescent model we assume all gene tree nodes are coalescences, and we associate to each node s on the species tree the number of extra lineages, which is the number of branches from the gene tree passing through s minus one. The number of deep coalescences $\text{ILS}(G_{i}^{r},S)$—also called ILS events—is the sum of extra lineages over all species tree nodes. Since we are using the LCA mapping these numbers will be minimal over all possible reconciliations—for more details, see Wu and Zhang (2011); Bansal and Eulenstein (2013). Because we do not know the root location of the gene trees, we apply the LCA mapping and calculate the minimum costs over all possible roots r to define the optimal root location. From the duplication-loss model we will then have $d_{1}(G_{i},S)=\text{DUPS}(G_{i}^{r^{*}},S)$ and $d_{2}(G_{i},S)=\text{LOSS}(G_{i}^{r^{*}},S)$ which will be the smallest amongst all r, and for the coalescent model we will have $d_{3}(G_{i},S)=\text{ILS}(G_{i}^{r^{**}},S)$, where the optimal root $r^{**}$ might be the same as $r^{*}$ or not.

As for the nonparametric distances, in theory they can be any estimate of disagreement between rooted or unrooted trees, of which the most popular example is the RF (or symmetric) distance (Robinson and Foulds 1981). Other examples include the SPR distance (Beiko and Hamilton 2006) and minimum weight matchings between tree branches (Nye et al. 2006; Bogdanowicz 2008). They are nonparametric in the sense that they do not try to model the biological reason for the disagreement, only the outcome. The RF distance can be calculated only when each leaf from one tree is mapped to at most one leaf from the other, restricting therefore the gene families that can be considered. However, very recently a generalization of the RF distance called mulRF was introduced that relaxes this constraint, allowing for one of the two trees in the distance calculation to have several leaves with the same label (Chaudhary et al. 2013). This multilabelled tree, or multree, is the gene tree. Although the original RF distance is between unrooted topologies, the RF distance can be used to compare a rooted tree to another one, rooted or unrooted (Day 1985; Górecki and Eulenstein 2012). Both the RF and the mulRF distances are calculated as the sum of bipartitions present in one tree but not in the other, but for the mulRF distance the species tree is extended such that all leaves representing a species labeled more than once in the gene tree are replaced by a multifurcation with same number of leaves as in the gene tree (Chaudhary et al. 2013). Therefore we have that $d_{4}(G_{i},S)=\text{mulRF}(G_{i},S)$, noting that this value is the same for any rooting of $G_{i}$ and S since we use the unrooted version of the trees. Like the reconciliation distances, the nonparametric ones ignore branch lengths, because they measure the disagreement between the topologies only.

### Sampling from the Posterior Distribution

Sampling directly from the above hierarchical model is very hard, so we employ a MCMC sampling (Liu 2001) where variables are updated one by one using Generalized Multiple-try Metropolis (GMTM) updates (Liu et al. 2000; Pandolfi et al. 2010). The GMTM algorithm for proposing an update of current state x to a new state y can be described as

1. Given current state x, draw k samples $y_{1},\ldots ,y_{k}$ independently from the proposal distribution p(·|x)
2. Calculate the weights ${\bar{w}}_{i}(y_{i};x)=\frac{w_{i}(y_{i};x)}{\sum_{l=1}^{k}w_{l}(y_{l};x)}$, where $w_{i}(y_{i};x)$ can be any bounded, positive function
3. Select $y=y_{j}\in y_{1},\ldots ,y_{k}$ according to ${\bar{w}}_{i}(y_{i};x)\;i=1,\ldots ,k$ and set $W_{y}={\bar{w}}_{j}(y;x)$ (assuming j is the chosen index)
4. Similarly, draw $x_{i}^{*}\sim p(\cdot |y)$ for i=1,…,j−1,j+1,…,k, and let $x_{j}^{*}=x$ (i.e., using same index j as before). Calculate ${\bar{w}}_{i}(x_{i}^{*};y)=\frac{w_{i}(x_{i}^{*};y)}{\sum_{l=1}^{k}w_{l}(x_{l}^{*};y)}$ and set $W_{x}={\bar{w}}_{j}(x;y)$
5. Accept $y_{j}$ as new state with probability
  (5) $$\min\left(1,\frac{\pi (y_{j})p(x|y_{j})}{\pi (x)p(y_{j}|x)}\frac{W_{x}}{W_{y}}\right)$$

where π(y) is the posterior distribution under y. A common choice for the weight w(y;x) is the posterior distribution π(y) (terms independent from y will cancel out). This is how the vector $\lambda_{0}$ is updated.

In the current implementation, when updating the gene trees we avoid direct calculation of the joint probability P(D,θ|G) of alignments D and models θ, as mentioned before. This joint probability P(D,θ|G) is assumed to be independent between gene families and can be expanded as the product of the phylogenetic likelihood and the prior of the substitution model (Yang and Rannala 1997; Larget and Simon 1999)

(6) $$P(D,\theta |G)=\prod_{i=1}^{N}P(D_{i},\theta_{i}|G_{i})=\prod_{i=1}^{N}P(D_{i}|\theta_{i},G_{i})P(\theta_{i})$$

We furthermore assume that for each gene family i we have a posterior distribution of trees $P(G_{i}|D_{i},\theta_{i})$ (e.g., estimated by some state-of-the-art Bayesian phylogenetic inference program) and use the posterior frequency of tree $\hat{G}$ as a resampling weight $g_{i}(\hat{G})=P(G_{i}=\hat{G}|D_{i},\theta_{i})\propto P(D_{i},\theta_{i}|\hat{G})$ (Smith and Gelfand 1992). This procedure is similar to the two-stage MCMC approach implemented in BUCKy (Ané et al. 2007), and in practice means that by using the resampling weights as the proposal distribution p(·|·) in the GMTM updates the terms $P(D_{i},\theta_{i}|G_{i})$ will cancel out. Under this approach the substitution parameters $\theta_{i}$ and the branch lengths are nuisance parameters and can be ignored. At every iteration we propose a new species tree by applying one or several branch swaps on the current one, and we also have a rerooting proposal that does not change the topological information.

### The Normalization Constant

When updating the species tree S or the distance penalty parameters $\lambda_{i\cdot }$, we must also update the normalization constant—also called the partition function (Murray et al. 2012; Chung et al. 2013). This constant is the denominator of our multivariate exponential distribution for tree distances, and ensures that this distribution sums up to one over all possible gene topologies with the same leaf set. It must be taken into account if we want to interpret probabilistically the resulting distances, and neglecting it can even change the relative score of species trees under some distances and parameter values (Bryant and Steel 2008). This function changes with the species tree and $\lambda_{i.}$, and computing it is impractical for gene trees with more than a few leaves. With the GMTM algorithm the weight function is not restricted to probability measures from the model. Hence we could postpone the partition function calculation to step (5) of the GMTM algorithm by employing the unscaled distance distribution as the weight function. However, the acceptance probability will depend on the ratio between the partition functions $Z_{i}(S^{*},\lambda_{i\cdot }^{*})/Z_{i}(S,\lambda_{i\cdot })$ which do not cancel out.

Our solution is to use an exchange algorithm, where the proposal of a parameter that affects the partition function is always accompanied by the proposal of an auxiliary gene tree, therefore, canceling out the partition functions (Atchade et al. 2008; Liang 2010; Caimo and Friel 2012; Murray et al. 2012). This auxiliary gene tree is an augmented variable which is unrelated to our resampled estimates—it is sampled through a secondary MCMC given the proposed $\left(S^{*},\lambda_{i\cdot }^{*}\right)$ and is discarded immediately after calculating the exchange ratio (Caimo and Friel 2010; Caimo and Friel 2012).

Namely, given the unscaled distance distribution $f_{i}(G,S,\lambda_{i\cdot })=e^{-\sum_{j=1}^{J}d_{j}(G,S)/(m_{ij}\lambda_{ij})}$ then the joint distance probability can be written as

(7) $$P(G|\lambda ,S)=\frac{\prod_{j=1}^{N}f_{i}(G_{i},S,\lambda_{i\cdot })}{\prod_{i=1}^{N}Z_{i}(S,\lambda_{i\cdot })}=\frac{f(G,S,\lambda )}{\prod_{i=1}^{N}Z_{i}(S,\lambda_{i\cdot })}$$

Then the GMTM exchange algorithm proceeds as follows, remembering that each state is composed of the species tree S and the vector λ, but in practice we update just S or just a block of $\lambda_{i\cdot }$ for a gene family i at a time.

1. Given current state x, draw k samples $y_{1},\ldots ,y_{k}$ independently from the proposal distribution p(·|x)
2. Calculate the weights ${\bar{w}}_{i}(y_{i};x)=\frac{w_{i}(y_{i};x)}{\sum_{l=1}^{k}w_{l}(y_{l};x)}$, where $w_{i}(y_{i};x)=f(G,S^{\left(i\right)},\lambda^{\left(i\right)})$ is the joint unscaled distribution
3. Select $y=y_{j}\in y_{1},\ldots ,y_{k}$ according to ${\bar{w}}_{i}(y_{i};x)\;i=1,\ldots ,k$ and set $W_{y}={\bar{w}}_{j}(y;x)$
4. Draw $x_{i}^{*}\sim p(\cdot |y)$ for i=1,…,j−1,j+1,…,k, and let $x_{j}^{*}=x$ Calculate ${\bar{w}}_{i}(x_{i}^{*};y)=\frac{w_{i}(x_{i}^{*};y)}{\sum_{l=1}^{k}w_{l}(x_{l}^{*};y)}$ and set $W_{x}={\bar{w}}_{j}(x;y)$
5. Assuming that the proposed state is $y_{j}=(S^{*},\lambda^{*})$, then draw a set of auxiliary gene trees $A=(A_{1},\ldots ,A_{N})$ from $P(\cdot |\lambda^{*},S^{*})$. This can be achieved by MCMC starting at the current G (notice that within this MCMC the partition functions cancel out.)
6. Accept $y_{j}=(S^{*},\lambda^{*})$ as new state with probability
  (8) $$\min\left(1,\frac{P(\lambda^{*}|\lambda_{0})p(x|y_{j})}{P(\lambda |\lambda_{0})p(y_{j}|x)}\frac{f(G|\lambda^{*},S^{*})f(A|\lambda ,S)}{f(A|\lambda^{*},S^{*})f(G|\lambda ,S)}\frac{W_{x}}{W_{y}}\right)$$

Importantly, our preliminary results suggested that point estimates from the posterior distribution of species trees are not affected by neglecting the partition function, at least when using multiple distances as we did. Therefore, to reduce the computational workload here we will show results where this normalization constant has been neglected.

### ML Estimation of Species Trees

We furthermore implemented a ML version of our model, where instead of sampling from the posterior distribution P(S,Θ|D) we try to find only the optimal values $\hat{S}$ and $\hat{\Theta }$ by incrementally decreasing the temperature kT of the distribution $P{\left(S,\Theta |D\right)}^{1/kT}$. Therefore, we can use the same algorithms as before, since as the temperature kT decreases only updates that improve the posterior distribution are accepted (Rubenthaler et al. 2009). The final state of this modified MCMC sampler is then its simulated annealing estimate, which can be interpreted as a ML supertree estimate under the arbitrary set of distances chosen by the user.

### *Software Implementation*: Guenomu

We implemented our Bayesian supertree model into the program *guenomu* (http://bitbucket.org/leomrtns/guenomu), where the user can choose which distances will be taken into account by the multivariate exponential distribution. In this manuscript, we work with two parameterizations: one considering only the reconciliation distances, which we will call the DLI model since it employs the minimum number of Duplications, Losses, and ILS; and another adding the mulRF distance, called the DLIR model. The program *guenomu* is parallelized at the gene family level, whereby the communication between computing nodes is minimized through the implementation of shared pseudo-random generation streams (Feng et al. 2003; Feng et al. 2006). It also allows for implementing other distances without compromising the model. Besides the files with the input gene tree distributions, the user just needs to provide a list with species names, under the assumption that the leaves from the gene families contain these species names. The mapping between each leaf from the gene trees and its corresponding species is then done automatically.

Besides sampling from the posterior distributions of genes and species trees, *guenomu* also outputs a formatted text file with the sampled continuous parameters together with the posterior probability (apart from a constant) for each iteration, after a burn-in period. This file can be used, among other things, in convergence diagnostics programs like *Tracer* (Rambaut et al. 2013) or *coda* (Plummer et al. 2006).

### Assessment of Accuracy Using Computer Simulations

To assess the performance of our implementation, we carried out a simulation study to compare *guenomu's* posterior species tree distribution with the true species trees and with other similar supertree methods described below. We generated 7089 replicate data sets using *SimPhy* (Mallo et al. 2014). This simulation environment takes into account the birth and death of (paralogous) loci inside a species tree as well as the coalescent process describing the gene tree within each locus , as in Rasmussen and Kellis (2012). *SimPhy* can take a species tree with information about effective population size and number of generations for each branch, and simulate the evolution of new loci through a birth–death process followed by the multispecies coalescent simulation of population samples (i.e, gene copies belonging to multiple individuals) inside each locus.

For each replicate data set, a species tree was generated under the Kingman coalescent process using the software Dendropy (Sukumaran and Holder 2010) such that the number of species was between 10 and 80 and the length from the root to tips was between $10^{2}$ and $10^{4}$ generations. We assumed that the effective population size was constant and that the species tree length in coalescent units was between 0.05 and 5, guaranteeing low to high levels of ILS (Leaché and Rannala 2011). Then we used *SimPhy* to simulate the evolution of a number of gene families inside each species tree, generating gene trees with branch lengths in substitution units. Input parameters for each replicate were sampled stochastically from the distributions summarized in Table 1. In short, for each gene family the number of individuals per species varied between 1 and 10 whereas the number of gene families ranged from between 2 and 50. Furthermore, each gene family differed from the species tree through a birth–death process of DL such that the average number of duplications was between $10^{-3}$ and 4. In the end, each replicate had gene trees with average sizes ranging from 35 to 1380 leaves. We furthermore assumed that different gene families from the same simulated data set can have distinct rates for the birth and death of new loci.

**Table 1.** Parameter values used in the simulations

	Description	Symbol	Distribution
Species tree			
*(Dendropy)*	Number of species		Uniform(10, 80)
	Number of generations (total tree height)		Uniform($10^{2}$, $10^{4}$)
	Expected number of duplications	$E_{dup}$	Uniform($10^{-3}$, 4)
	Number of gene families	N	Uniform(2, 50)
	Rate heterogeneity multiplier	$H_{s}$	Gamma(1, 1)
Locus tree^a^			
*(SimPhy)*	Gene duplication rate^b^	β	Exponential($E_{dup}/\sigma$)
	Gene loss rate		Uniform(0, 0.75×β)
	Rate heterogeneity multiplier	$H_{l}$	Gamma(1, 1)
Gene tree^a^			
*(SimPhy)*	Effective population size	$N_{e}$	2000× LogNormal(0, 0.25)
	Number of individuals per species ^c^		Uniform(1, 10)
	Substitution rate (per time unit)		0.001
	Rate heterogeneity multiplier	$H_{g}$	Gamma(1, 1)
Gene tree			
Uncertainty	Maximum number of generated trees		160
(in-house)	Tree dispersion term	$L_{T}$	Uniform(2, 5)
	Tree location term	$D_{T}$	Uniform(3, 6)
	Frequency of trees with uncertainty	$p_{T}$	1.1× Beta($L_{T}\times D_{T}$, $D_{T}$)
	Branch location term	$L_{B}$	Uniform(1, 5)
	Branch-wise error probability	$p_{B}$	1.5× Beta($L_{B}$, 1)

Notes: Each simulation replicate was parameterized with values sampled from predefined statistical distributions. Given a species tree, simulated with *Dendropy*, the program *SimPhy* was used to simulate gene family trees. An in-house program was then used to mimic gene tree uncertainty, transforming each gene tree in a distribution of gene tree topologies in an attempt to emulate the effect of alignment-based phylogenetic inference in practice.

^a^ terminology used by *SimPhy*

^b^ the term σ is the total sum of branch lengths (in generations) in the species tree

^c^ common for all gene families, emulating perfect sampling of individuals

Both the generated species tree and each gene family tree were initially ultrametric, with lengths given in generation times. But we used *SimPhy* to further simulate substitution rate heterogeneities at several levels, by applying gamma-distributed rate multipliers at the branches of each of the simulated trees from the three-tree model, namely the species tree, the locus tree, and the gene tree (Mallo et al. 2014). As a result, the gene family trees did not follow a molecular clock. Although, currently our model for species tree inference do not take into account this branch length information, it is relevant to simulate gene tree uncertainty (i.e., tree inference error), as we will see below.

### Gene Tree Uncertainty

The simulation procedure just described provides us with the single, true tree underlying each gene family. However, our model explicitly considers gene tree uncertainty, relying as input on a sample of the posterior distribution of trees for each gene family. To obtain these we could simulate sequences along the true gene trees (Yang 2007; Fletcher and Yang 2009), and then infer posterior gene tree distributions using popular Bayesian phylogenetics programs like MrBayes (Ronquist et al. 2012), PhyloBayes (Lartillot and Philippe 2004) or BEAST (Drummond et al. 2012). Unfortunately this “parametric” approach is extremely slow, limiting the scope of our simulations, so we carried out only a small simulation experiment under this strategy (see section below). Instead, for our main simulation study we devised a nonparametric way of directly building gene tree distributions for each gene family in an attempt to recreate gene tree uncertainty.

For each true gene tree we generated a population of topologies that may differ from the original by one or more nearest-neighbor interchanges (Waterman and Smith 1978), assuming a probability $p_{T}$ of applying branch swaps to each tree, and a probability of branch swap per branch inversely proportional to its length, bounded by the maximum swap probability $P_{B}$. The latter accounts for the observation that shorter branches are harder to reconstruct, and generally are where most of the uncertainty in the phylogenetic estimation is concentrated. A branch swap means that a bipartition on the tree is replaced by one of its two alternatives. In this way, a proportion $\left(1-P_{T}\right)$ of resulting trees are expected to be identical to the original gene tree, whereas the others should differ by at least one branch. Values for $P_{B}$ larger than 1 are allowed, meaning that even larger branches will have high uncertainty. For low levels of uncertainty the most frequent topologies will match the true gene family trees, although for the parameter values employed in our simulations almost all data sets contained at least one most frequent gene tree distinct from the true one. The pseudocode for applying uncertainty to a gene tree is described in the Appendix.

**Figure 1.**
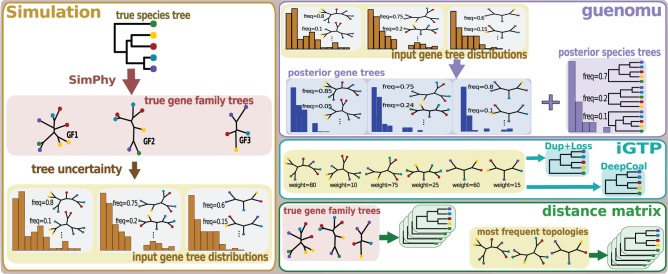
Simulation workflow. On the left a single data set is produced using *Dendropy* and *SimPhy*. The true species tree (rooted, with branch lengths) simulated by *Dendropy* is used as input by *SimPhy* to generate several (rooted) phylogenies, one per gene family. Then, uncertainty is added such that we have a distribution of topologies (unrooted, no branch lengths) per gene family. These collections will be used as input to the inference programs in the right panel. Our software *guenomu* estimates the posterior distribution of species trees (rooted, without branch lengths) and the posterior distributions of gene family topologies, based on all input gene tree distributions. The software *iGTP* also uses the input gene trees after transforming the frequencies into integer values representing weights, and estimates two rooted species trees: one under the Duplication and Loss cost, and another under the Deep Coalescence cost. For the distance-based species tree inference algorithms only one tree per gene family is used, and two alternative choices were attempted: one was to use the true gene families, with branch lengths; and the other was to use the most frequent gene trees (topologies only) after introducing phylogenetic uncertainty. In the later case it was assumed that all branches had the same length.

The general workflow of the simulation study is represented in Figure 1. Each replicate data set was composed by a species tree and a set of 2–50 gene families, where phylogenetic uncertainty around each gene family is represented by a tree collection with up to 160 distinct topologies. As a result of the introduction of uncertainty, in 6% of simulations all gene tree topology distributions showed as the most frequent tree the original, true tree. In the remaining 94% at least one distribution had a gene tree more frequent than the true tree. From these, wrong trees had on average a cumulative frequency 17% higher than the true tree, although in all cases the true topology could be found amongst the input gene trees, even if at a low frequency. These gene tree frequencies were assumed by *guenomu* to be the resampling weights (posterior frequencies from independent analysis) of each tree.

### Simulations with INDELible and MrBayes

To estimate whether the above procedure was a good approximation to the empirical estimation of gene trees from biological sequences, we also employed a parametric simulation of input gene tree distributions for a few scenarios: given the true gene family tree generated by *SimPhy*, we used *INDELible* (Fletcher and Yang 2009) to generate an alignment from which the posterior distribution of trees was sampled by *MrBayes* (Ronquist et al. 2012). For *INDELible* we simulated an alignment of $10^{3}$ amino acid sites under the WAG model (Whelan and Goldman 2001) and without indels where the total tree length was rescaled to 1 (such that each site will have on average one replacement). Two independent runs of four chains each (one cold and three heated) were simulated for $5\times 10^{5}$ iterations by *MrBayes* using the true WAG model.

Due to heavy computational requirements of MrBayes for large data sets (Supplementary Fig. SF6 available on Dryad; http://dx.doi.org/10.5061/dryad.74922), we constrained the simulation parameters: each replicate data set had at most 10 gene families, with up to two individuals per species, and with no more than 50 species. Therefore a typical data set had gene families with an average of 33 leaves, and in total 1362 replicates were generated under this parametric approach. Unless otherwise stated, our results refer to the nonparametric simulation of gene tree uncertainty.

### Comparison with Other Species Tree Approaches

For each simulated data set two Bayesian *guenomu* runs were conducted for $2\times 10^{5}$ iterations each, under both the DLI and DLIR parameterizations. The number of iterations was decided based on the apparent convergence of the posterior probability sequences of a few samples, using the *coda* library for R (Plummer et al. 2006). We also did a preliminary analysis with the mulRF parameterization only, but pilot experiments showed bad performance and we did not explored it further. Each *guenomu* run generates a (posterior) distribution of rooted species trees, and their equivalent posterior distribution of unrooted trees. We used as point estimates of this distribution both the *maximum a posteriori (MAP) tree* and the 50% majority rule *consensus tree*—remembering that they contain only the topological information and no branch lengths. Furthermore, under the DLIR model we conducted another run of our model to estimate a ML species tree using simulated annealing, as described above. Each *guenomu* run took on average 1.5 hours to complete, using the serial version on a single processor. Most runs completed in less than 3 hours, with the longest data set taking 9 hours to finish (see Supplementary Fig. SF1 available on Dryad; http://dx.doi.org/10.5061/dryad.74922).

For the same simulated data sets, and for comparison purposes, we also employed several other supertree-related models to reconstruct the species tree, namely GTP (Bansal and Eulenstein 2013) and coalescent distance matrix methods (Liu et al. 2009a), also called coalescent summary statistics methods (see below). Because in this study we consider gene trees evolving inside a species tree under duplications, losses, and ILS, we did not consider species tree methods that cannot deal with multilabelled gene trees, like the supermatrix approaches or consensus methods like those implemented in BUCKy (Ané et al. 2007).

GTP aims to find species tree that minimizes its total reconciliation cost with a given collection of gene tree topologies. Here we used the software *iGTP* (Chaudhary et al. 2010), which assumes that the disagreement between gene trees and the species tree is either due to DL, or to ILS. An independent analysis was conducted under each assumption. *iGTP* allows for each gene tree to have a weight associated with it, and therefore we used the topology frequencies resulting after the introduction of phylogenetic uncertainty (see above). However, for computational feasibility we limited the input gene trees to the most frequent quartile, which should inflate its accuracy compared with using all samples.

We implemented in-house several summary-statistic coalescent species tree reconstruction methods based on gene-wise distance matrices, as described in Helmkamp et al. (2012). This class of methods start by creating a matrix of distances between species for each gene phylogeny. Such matrices are then merged into a single one, from which a species tree is built using a clustering algorithm. The four procedures implemented were GLASS (Mossel and Roch 2008), STEAC (Liu et al. 2009b), SD (Maddison and Knowles 2006), and MAC (Helmkamp et al. 2012), which differ on how they handle contradicting distances for the same pair of species. This distinction stems from the fact that when there is more than one individual from the same species in a given gene family, its tree will have more than one path for the same pair of species. In such cases the GLASS and SD methods will choose the minimum distance between the pair as the element gene-wise distance matrix, whereas the MAC and STEAC methods will take the average between all distances for the same pair of species. However when there are several gene families each matrix might have a distinct distance between a given pair of species. In this case, the GLASS and the MAC methods use the minimum value, whereas the SD and STEAC use the mean distance across matrices. The species tree is then reconstructed based on this final distance matrix, where SD and STEAC employ an UPGMA, and GLASS and MAC use a single-linkage clustering algorithm (Helmkamp et al. 2012).

These distance matrix methods are grounded on the divergence times between species, and thus rely heavily on the individual branch lengths. Moreover, these methods assume a single gene tree for each gene family. We used the true gene trees with the true branch lengths as input to these methods, since those are available to us. However, this is not a fair comparison with *guenomu* and *iGTP* since for these approaches we assumed that there is gene tree uncertainty. To make a fair comparison, we in addition estimated species trees with these methods but using only the most frequent gene topology from the input gene tree distributions, assuming that all branches had a length of 1.0 (since our input distributions contain only the topologies but no branch lengths). The latter corresponds to using the path length (number of nodes) between leaves as the pairwise distances (Steel and Penny 1993). From each of these methods we obtained a single estimate of the species tree.

Due to their computational complexity, we did not evaluate sequence-based approaches like *BEAST (Heled and Drummond 2010) as each simulated gene family had hundreds of taxa, on average. Indeed, it would have been very interesting to compare our model with the recently developed software Phyldog, which implements a probabilistic model to estimate the ML species tree given a set of gene families (Boussau et al. 2013). Unfortunately in our experience this software is very difficult to install and run, and we were not successful in designing a pipeline that could systematically execute Phyldog with our simulated data sets.

### Performance Measures

We calculated *tree accuracy* as the number of splits (bipartitions or branches) from the true unrooted species tree that were successfully recovered in the inferred species tree, divided by the total number of splits on the true unrooted species tree. This accuracy measure will go from zero when no true splits are recovered to one when the true species tree topology is completely recovered. We also recorded the proportion of simulations where the true unrooted species tree was perfectly recovered (i.e., the proportion of replicates where tree accuracy was one, or *true tree recovery*.) In addition, from the analyses where we had a sample from the posterior distribution we also calculated the proportion of credible sets (at the 95% level) that contained the true unrooted species, or *true tree coverage*.

### Empirical Benchmark

To test our model on real data, we downloaded all ML gene family trees from the TreeFam database (Schreiber et al. 2014) and then pruned all data outside the 12 *Drosophila* species subtree. We considered all the gene families with more than 3 species. Within these we identified 4591 single-copy gene families (i.e., just orthologs), and from the remaining 2562 gene families with at least one paralog, we arbitrarily selected the 43 largest ones, with number of leaves between 102 and 295. As in the simulations, we added phylogenetic uncertainty around the TreeFam gene families as described above, generating a distribution of gene trees for each gene family.

## Results

### Species Tree Aaccuracy

**Figure 2.**
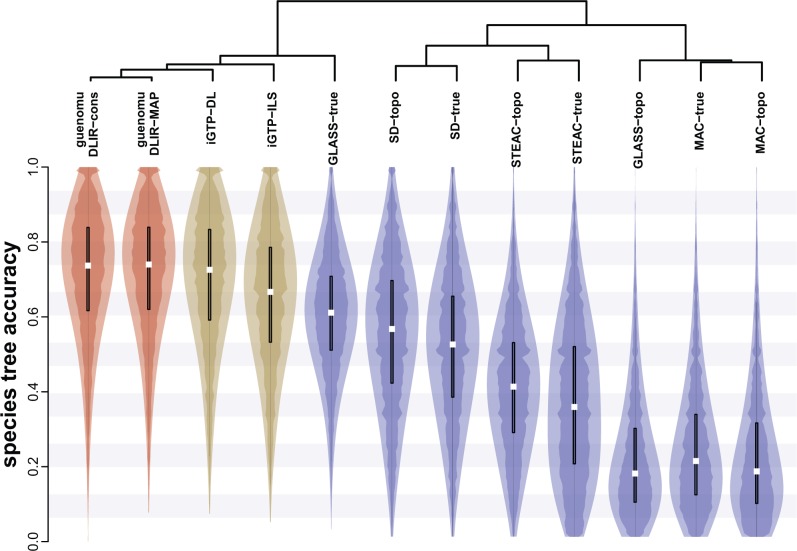
Species tree accuracy of several inference methods. Each violin plot (kernel density curves plus a boxplot) represents the distribution of tree accuracy values for the different methods evaluated. From each posterior distribution estimated by *guenomu* under the DLIR parameterization we obtained two point estimates of the species tree: the MAP tree and the consensus tree (labeled ‘MAP’ and “cons”). We also obtained two estimates of species trees using *iGTP*, running the program under the DL cost and under the ILS cost (“*iGTP*-DL” and “*iGTP*-ILS”). We also ran four distance matrix approaches (GLASS, SD, STEAC, and MAC) using two types of data sets, just the topologies with uncertainty (‘topo’) or the true simulated gene family trees with branch lengths (“true”). At the top, we show the hierarchical clustering of the different methods based on their tree accuracy values for all replicates.

In the simulation scenarios considered, *guenomu* performed best regardless of its parameterization, showing median tree accuracies around 0.7 (Fig. 2). In fact, we could not distinguish between the species tree accuracies estimated through the DLI and DLIR parameterizations (Wilcoxon Signed-Rank P>0.5). *Guenomu* was however followed very closely by *iGTP*, which was slightly more accurate with DL costs than when ILS costs were used. The average increase in accuracy between *guenomu* and *iGTP* was of only 1% when considering all replicates, and of 2% when considering only data sets with more than 30 species under the DL cost for *iGTP* (the increase was of 6% when compared to the ILS version of *iGTP*). However, these differences were highly significant using a paired-difference test (one-tailed Wilcoxon Signed-Rank $P<10^{-15}$). *Guenomu*'s ML estimates were very similar to *iGTP* under DL cost (KS test P>0.5), however, slightly more accurate according to a paired test (one-tailed Wilcoxon Signed-Rank $P=2\times 10^{-3}$). When given the true trees, the GLASS method ranked next, but performed badly when gene trees were uncertain. The SD method did worse, performing equally well on true and observed gene family trees. By far, the worst methods were STEAC and in particular MAC, even when the true gene trees were given.

**Figure 3.**
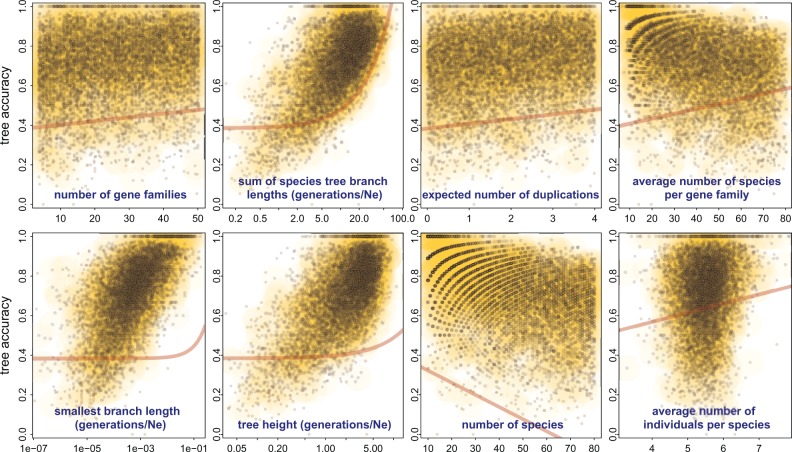
*Guenomu*'s tree accuracy with respect to simulation parameters. Here, the species tree was estimated as the consensus tree from the posterior distribution assuming a DLIR parameterization. Based on a multiple linear regression analysis, these are the parameters that most significantly affected tree accuracy: at the top we have, from left to right, the total number of gene families, the total species tree length (in coalescent units), the expected number of duplications per gene family and the average number of species represented by gene family; at the bottom we have the length of the smallest branch in the species tree (in coalescent units), the height of the species tree from root to tips (scaled by the effective population size), the total number of species on the species tree and the average number of individuals from same species per gene family. Over each panel we show the regression line overlaid.

To better understand the effect of different parameter values used in the simulation, we fitted a multiple linear regression model to the tree accuracies obtained with *guenomu*'s consensus tree under the DLIR model (Fig. 3). Several explanatory variables were significantly and positively correlated with tree accuracy (P-value $<10^{-15}$ unless otherwise stated), including the sum of species tree branch lengths in coalescent units, the expected number of duplications per gene family on the species tree, the average number of individuals from the same species per gene family, and the average number of distinct species per gene family (P-value $<10^{-8}$). The species tree height (length from root to tips) and the smallest branch length, both in coalescent units, were also significantly correlated with tree accuracy with P-values, respectively, smaller than $10^{-9}$ and $10^{-4}$. In any case, the most influential parameter was apparently the number of species, which was negatively correlated with tree accuracy (P-value $<10^{-15}$).

**Figure 4.**
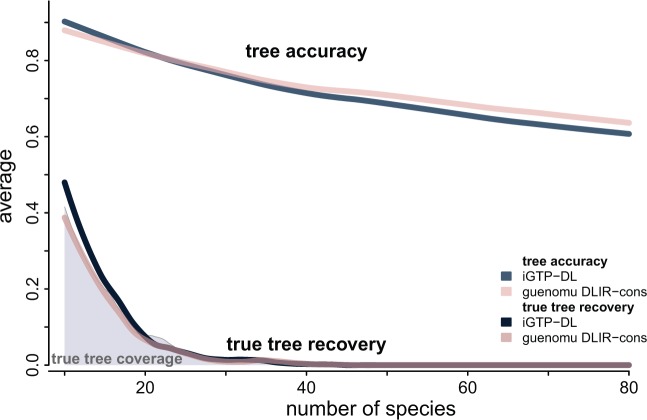
Tree accuracy and true tree recovery for *guenomu* and *iGTP* according to the number of species. The lower lines show the true tree recoveries pooled over the number of species, whereas the higher lines show the average tree accuracy under different conditions. The gray area represents the true tree coverage under *guenomu* DLIR model (i.e., the proportion of data sets where the true species tree was within the 95% credible set). Each line is actually a smooth regression line over the individual values. For *iGTP* the species tree was estimated under the DL cost, whereas for *guenomu* we plot the Bayesian consensus under the DLIR parameterization.

**Figure 5.**
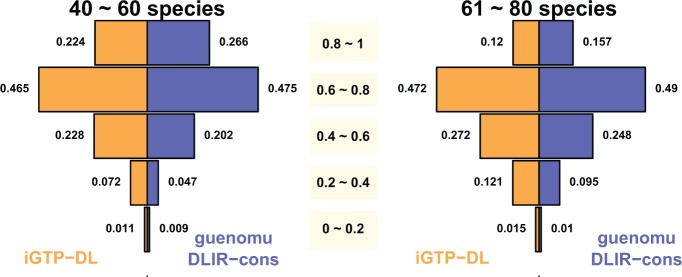
Tree accuracies of *iGTP* and *guenomu* for large data sets. On the left we have all data sets where the number of species was between 40 and 60, and on the right we have the pooled replicates with species tree sizes between 61 and 80 leaves. Each bar represents the proportion of data sets with tree accuracies between the ranges described in the middle, such that at the top we have the fraction of simulations where more than 80% of the splits were recovered, while the inferences whereas less than 20% of the true splits were found are the bottom.

Neither *guenomu* nor *iGTP* were able to recover the true species tree for more than 40 species (Fig. 4). Under *guenomu*, the true tree could be found within the 95% coverage on data sets with up to 40 species. Nonetheless, even when the true species tree could not be found, *guenomu* found trees consistently more accurate than *iGTP* for large trees (Fig. 5). In these cases, the consensus tree from the posterior samples under *guenomu* was in any case the best estimate. For example, for the simulations with more than 60 species, 64% of the species trees reconstructed from *guenomu* found at least 60% of the true splits, against only 59% of the trees inferred by *iGTP* (Fig. 5). About 15.7% of trees from guenomu reconstructed more than 80% of the true splits for the same large data sets, against 12% when *iGTP* was used.

To obtain more details on this slightly increased performance of *guenomu*, we fit another multiple linear regression model over all replicates where the dependent variable was the difference in tree accuracy between *guenomu*'s consensus estimate from the DLIR model and *iGTP* under the DL cost. This indicated that *guenomu* significantly outperformed *iGTP* (p-values $<10^{-5}$) for data sets with more and larger gene families, with shorter species trees (as measured by the sum of its branch lengths in coalescent units). The improvement was also significant (P-values $<10^{-3}$) for MAP input gene trees with low frequency and for lower levels of gene tree uncertainty as measured by $P_{T}$.

### Simulations with INDELible and MrBayes

Similar performance trends were observed for the small data sets where we simulated sequences with INDELible and obtained posterior gene distributions with MrBayes, although the accuracies were overall lower (Supplementary Fig. SF3 available on Dryad; http://dx.doi.org/10.5061/dryad.74922). This may be a reflection of the lower accuracy in the input gene tree distributions themselves, as estimated by *MrBayes* when compared to our nonparametric algorithm (Supplementary Fig. SF2 available on Dryad; http://dx.doi.org/10.5061/dryad.74922). Furthermore the simulation scenarios under which we could apply parametric gene tree uncertainty—few gene families with small number of taxa, spanning fewer species—are those where by chance the advantage of *guenomu* is less visible, as described in previous section.

### Gene Tree Accuracy

**Figure 6.**
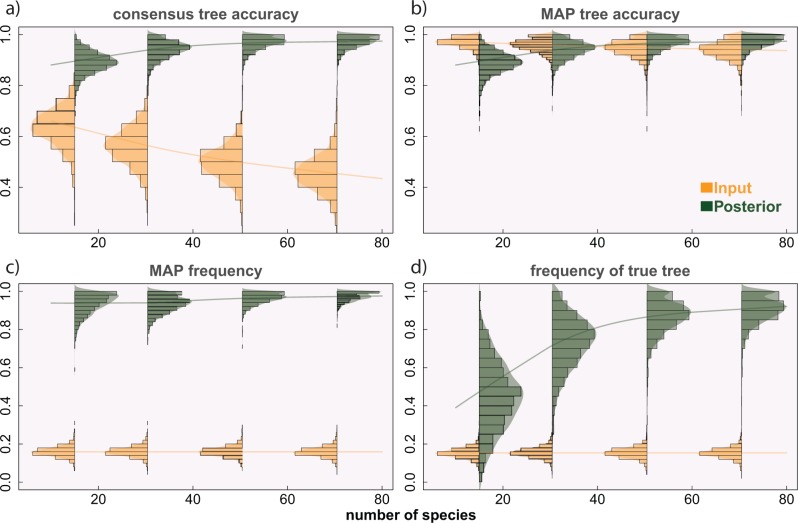
Input and posterior gene tree distributions. Each panel shows the distribution of input gene trees (after generation of tree uncertainty using our algorithm) and their posterior counterparts (resampled by *guenomu*) for several ranges of species tree sizes, together with a smooth regression line over all samples. The panels at the top show the accuracies of the consensus (a) and MAP (b) estimates, when compared to the true gene trees simulated by *SimPhy*, whereas the bottom panels display the frequencies of the MAP (c) and true (d) gene trees. All values are averages over all gene families from each replicate.

Our software *guenomu* not only estimates the distribution of species trees but also resamples the input gene trees, providing a posterior distribution of gene trees for each gene family. For each replicate, we compared the input gene tree distributions generated nonparametrically with their posterior gene tree distributions output by *guenomu* (Fig. 6). Compared with the original input gene tree distributions, *guenomu*'s gene tree posterior distributions included the true tree most frequently, besides resulting in much more accurate consensus trees, and in more accurate MAP trees when the number of species was larger.

The main difference between our tree uncertainty algorithm and the parametric approach (*INDELible* + *MrBayes*) was the distance between the consensus and MAP estimates (Supplementary Fig. SF2 available on Dryad; http://dx.doi.org/10.5061/dryad.74922). With our method the consensus tree of each gene family was much less accurate than the MAP tree, reflecting the presence of a lot of noise around the true tree that, however, was always present. Under the more realistic simulation using *MrBayes* both the consensus and MAP estimates had similarly good accuracies, even when the true tree was not included in the sample of gene trees. Therefore, the input gene tree distributions described here seem to be a good, although imperfect, proxy to posterior gene tree distributions estimated by Bayesian phylogenetics methods.

For input gene tree distributions generated parametrically, *guenomu* was similarly capable of reducing the uncertainty from the gene tree distributions, with the caveat that we did not observe the increase in the consensus gene tree accuracies estimated by *guenomu* for larger data sets (Supplementary Fig. SF2 available on Dryad; http://dx.doi.org/10.5061/dryad.74922).

### TreeFam Drosophila Gene Families

As for the TreeFam data, *guenomu* reconstructed the known *Drosophila* species tree (Stark et al. 2007) as well as the root location for the data set with few large gene families (43 genes with more than 100 members each). The correct root location could be found even in the absence of an outgroup. The rooted species tree estimation from this data set was robust to gene tree uncertainty. That is, even after replacing each ML tree by a distribution of topologies as described before, *guenomu* was capable of finding the known species tree with posterior probability equal to one.

However, for the large data set composed of single-copy gene families (4591 gene families with up to 12 members each), although the unrooted topology could be found in the absence of gene uncertainty, the rooted location was not correctly inferred. In the presence of gene uncertainty, even the reconstructed topology missed one or two splits.

## Discussion

### Reconstructing Species Trees from Gene Families with Lineage Sorting

Most phylogenomic analyses start with the identification of ortholog sequences, after some process of sequence collection and pruning of paralogs (e.g., Koonin and Wolf 2008; Dunn et al. 2008; Medina et al. 2011; Sanderson et al. 2011; Bininda-Emonds 2011; Williams et al. 2012; Lang et al. 2013; Romiguier et al. 2013; Salichos and Rokas 2013). Much less often, alternative species tree analyses focus on gene duplication and loss (Katz et al. 2012), or consider paralogs at all (Holton and Pisani 2010). The common practice of removing data (sequences or whole genes) before analyzing the reduced data sets is sometimes needed, in order to conform to model assumptions or due to computational limitations of the methods. This process of data selection is also rooted on the elusive idea of removing uncertainty and improving resolution, which is anathema to the Bayesian paradigm. Here we propose a Bayesian supertree model that does not need to separate homologs from paralogs, that can work with multiple gene families and that is also able to deal with multiple individuals per species. It also minimizes the pre-processing of data to conform to strict standards of resolution, since the model can cope with uncertainty in the phylogenetic inference. Therefore our approach, implemented in the program *guenomu*, is able to work with all the available data at hand.

Our simulations suggest that *guenomu* is able to reconstruct reasonably accurate species trees in the presence of gene duplications, losses, and deep coalescences, even though right now it uses relatively simple distances that do not take into account branch lengths. Although this is not a panacea, it suggests that exploring these apparently naïve models of disagreement may be an attractive alternative to biologically realistic frameworks, specially once we aim for a consilience of methods. We hope that more analyses are conducted under broadly usable frameworks like the one we offer, at least as a starting point before embarking into other ones with additional assumptions.

Both DLI and DLIR parameterizations of *guenomu* performed almost identically in recovering the species tree, but the DLIR model, which includes the mulRF distance (Chaudhary et al. 2013), helped to reduce the uncertainty from the posterior gene tree distributions. This might be due to the “coarseness” of the distances employed: even when there are several gene trees equidistant to the species tree under a given metric, they may be distinguishable under another metric. Since the computational overhead of the DLIR parameterization over DLI is minimal, it seems worthwhile to use DLIR. The similarity of posterior distributions between the two parameterizations for each replicate suggests the convergence of the chains, but we recommend the use of proper convergence diagnostics (Rambaut et al. 2013; Plummer et al. 2006) based on independent runs for real analyses using empirical data sets. The user can also decide how the initial state of the chain is chosen based on an annealing algorithm—by “heating” the chain before sampling, we can ensure that the original states are random, and thus accurate convergence analyses can be pursued.

In the simulations, the true species tree became more and more difficult to recover as the number of species increased. However, even in these cases the majority of true splits were recovered through the consensus or MAP estimates, suggesting that our model can be applied to relatively large species trees. We notice however that for the largest species trees the true species tree was not covered in the 95% credible interval. We also applied the same analysis for species trees simulated under a pure-birth (Yule) process, obtaining better accuracies even for a large number of species (Supplementary Fig. SF4 available on Dryad; http://dx.doi.org/10.5061/dryad.74922).

As expected, the length in coalescent units of the smallest branch of the species tree and the overall species tree length had a great influence on species tree accuracy, together with the number of species. Indeed, we expect more ILS and consequently more disagreement between gene and species trees next to short branches, and also over a short generation span within large effective population sizes. In Figure 3, the simulated smallest branches on the species tree comprise scenarios with high probability of ILS, once we recall that the probability of failure to coalesce for two nodes inside a branch of length t (in coalescent units) equals $2/3e^{-t}$ (Hudson 1983). The range of species tree heights scaled by the effective population size is also concentrated around values smaller than $4N_{e}$, considered “difficult demographic scenarios” (Leaché and Rannala 2011). Species trees simulated under a pure-birth process show less variability between branch lengths than those simulated under the coalescent, and a comparison of *guenomu*'s performance between data sets simulated under both scenarios confirm the considerations above (Supplementary Fig. SF5 available on Dryad; http://dx.doi.org/10.5061/dryad.74922). Under the conditions simulated, our model performed better on data sets with more and larger gene families, but representing fewer species. Small gene families over a large number of species proved more challenging to the methods compared.

Surprisingly, *iGTP* also provided good species tree estimates, being significantly but not egregiously worse than *guenomu* as the number of species increased. We did not expect this behavior because *iGTP* cannot handle both DL and ILS costs at once, and nonetheless assuming just a DL cost seemed to work quite well even in the presence of moderate ILS. *iGTP*'s performance decay for larger species trees might be due to the increase in ILS (as indicated by our linear regression results), when *guenomu* is favored. We also notice that for *iGTP* we used the top 25% most frequent gene trees from each input distribution, using frequencies as weights, which gave better results than using only the most frequent trees or all trees (results not shown). This is likely due to the long tail of wrong trees generated by our tree uncertainty algorithm. Indeed, *iGTP* is faster than *guenomu*, which then makes it a very competitive approach. On the other hand, *guenomu* can replicate completely *iGTP*'s functionality through its ML algorithm and has all the advantages of Bayesian methods, which could furthermore work with other complex distance costs in the future.

The coalescent distance matrix methods evaluated here assume that the source of disagreement comes from ILS, therefore, it comes at no surprise that they did not perform well under our simulations, with an important contribution from DL. DL, when unaccounted for, will have a detrimental effect on the estimated coalescent process (Rasmussen and Kellis 2012). Although this comparison might seem somehow unfair to these summary statistics methods, it serves also as a warning against using attractive algorithms without considering their assumptions. It might be interesting, however, to explore more extensively these methods and see how they fare against *guenomu* or *iGTP* when there is only ILS.

We also observed that both *iGTP* and *guenomu* benefit from large gene families, with several representatives from each species—even though we do not distinguish paralogs or populational samples. This is evidence against the trend of finding good, hand-crafted sets of orthologs at the expense of the paralogs that must be discarded from the analysis. This pruning may be done after the complete data set was used to create the posterior distribution of species trees, in a subsequent analysis, but not before.

The algorithm we devised to simulate gene tree uncertainty allowed us to explore a wider range of simulation scenarios without compromising the conclusions. The inferred species trees under the parametric simulation (*INDELible* + *MrBayes*) were usually less accurate in this case, but similar performance trends were observed.

The root location of the *Drosophila* largest gene families was found even without an outgroup since the presence of paralogs is informative about the optimal location of duplication nodes (Katz et al. 2012). For the single-copy genes data set, on the other hand, the root location was not correctly inferred for the same reason: orthologous data sets lack such phylogenetic information. Furthermore, the smaller gene families were more sensitive to the effect of tree uncertainty.

### Advantages and Shortcomings of Our Bayesian Supertree Method

Bayesian methods in general give us information about the reliability of their findings through the posterior distribution. The flatness of the posterior distribution of species trees, or the frequency of the most frequent topology can give us a hint about the amount of signal contained in the data. The use of a Bayesian framework not only allows for model selection, but also facilitates the creation of models of increased complexity, subject always to computational constraints. Thus, our framework can be extended in a straightforward manner to handle more complex situations, and indeed incorporating more biological realism. Our distinct parameterizations allow us to compare models that use different sets of distances, such that we can, in principle, compare their underlying assumptions.

Our modeling of “phylogenomic error” can be seen as a generalization of optimal Bayes estimators, like the consensus tree or quartet puzzling (Huggins et al. 2011). Such a model has already been suggested as an alternative to the detailed description of the processes causing the tree incongruences, whereas at the same time maintaining the convenience of supertree methods (Steel and Rodrigo 2008; Cotton and Wilkinson 2009). This class of methods can help bridge the gap between sequence-based alignment and phylogenomic analyses (Cotton and Wilkinson 2009), and ours is the first implementation of this class of methods.

Originally, parsimonious reconciliation methods under the DL or the ILS costs worked with rooted gene trees and rooted species trees (Guigó et al. 1996; Than and Nakhleh 2009). However, because we define the DL and ILS distances as the minimum cost over all possible gene root locations, our model works with unrooted input gene trees. This is an important advantage, as in in real life often there is no reliable information about the rooting at the gene tree level, and fixing a root location can severely influence the resulting inferences.

The current implementation of our model uses only the topological information, ignoring branch lengths, but it can be extended in the future to deal explicitly with branch lengths on the gene and species trees. However, it is possible to use crudely the branch lengths as an indication of confidence in the bipartition, as implemented in our generator of gene tree uncertainty.

Other topological distances can be easily implemented as well, taking advantage of our solution to the normalization constant. An example of a promising distance would be one based on the recently developed DLCpar algorithm (Wu et al. 2014), which can find the most parsimonious reconciliation scenario by considering duplications, losses, and deep coalescences at once. Another option would be based on algorithms for finding the minimum cost reconciliation in the presence of duplications, losses, and HGT (Tofigh et al. 2011; Doyon et al. 2011). One drawback is that many of these algorithms do not return a single solution, since under these scenarios the best reconciliations are not unique. We have already added an approximate SPR distance (de Oliveira Martins et al. 2008; de Oliveira Martins and Kishino 2010) into our multivariate exponential distribution, to model the effect of HGT between the gene and species trees. Unfortunately, however, this approximation does not work with multrees, as is the case with many other distances. Therefore, this extension is left as an experimental feature of *guenomu*, that might help for cases where HGT cannot be neglected.

It has been shown that the normalization constant can have an important effect on the species tree estimation in a probabilistic setting when the RF distance is used Bryant and Steel (2008), and it certainly can affect other metrics. In our context, it means that some species trees are naturally closer than others to the space G of gene trees for a given gene family, being then spuriously chosen. However, our simulations on small data sets did not show any apparent differences in the estimated species trees whether or not we include its estimation (results not shown). We believe this is due to our usage of several distances, since they may even out the sum over G. As a result it becomes less likely that a given species tree $S_{1}$ is favored over another $S_{2}$ due to the ratio $Z(S_{1},\lambda )/Z(S_{2},\lambda )$ despite being more dissimilar, since $S_{1}$ would have to be closer to all G∈G according to all distances. It is not unreasonable to expect that a species tree $S_{1}$ favored under one distance may be disfavored under another distance, like for instance the DL and the ILS costs. Since the calculation of the normalization constant requires a secondary MCMC for each iteration, we decided not to use it in our reported simulations (that are centered around point estimates of the species tree), speeding up our inference by around 10 times. However, even if it does not influence the estimated consensus or MAP trees, it can hamper other statistical interpretations, like the estimation of credible intervals or calculation of the marginal likelihood. Therefore, whenever enough computational resources are available the normalization constant should be included in the analysis.

One might worry about a possible correlation between the distances used that might affect the performance of our method. We have seen that this is not the case since our model performs better than *iGTP* where distances are used in isolation. Furthermore, these distances are not equivalent as shown by distinct results using the DL or the ILS costs under *iGTP*. Indeed, the distances used are not redundant (Zheng and Zhang 2014), but even the inclusion of a distance partially correlated with an existing one should not be alarming, as long as there are cases where it can add new information. Actually, even a distance that is completely equivalent to another metric should not bias the performance, with the only drawbacks of wasting computing time and increasing the variance over their penalty parameters (since the model can not distinguish between them). This concern makes more sense in a pure parsimonious approach, where the weights for the costs must be decided beforehand, in which case we might want to give lower weights to groups of related distances. Each term in the multivariate exponential distribution acts as a penalty against dissimilar gene/species tree pairs, and therefore similarity according to only one metric does not guarantee a high probability for the given gene/species tree pair.

When using resampling weights it is important to have a large sample with all reasonable trees represented for each gene family (Smith and Gelfand 1992), specially since even lower likelihood trees might contribute more to the species tree than the ML estimate (Boussau et al. 2013). Indeed, the presence of the true gene tree amongst the samples improved performance, as was the case when we compared our nonparametric gene tree uncertainty generator with the parametric simulations using MrBayes. Therefore, ideally under our importance sampling algorithm we should avoid using point estimates of the gene trees (like the ML gene tree, for instance), although for the TreeFam data set it did not hamper the analysis. Nonetheless this simplification allows us to focus on the distribution of tree distances while using well-established software to estimate the individual gene tree distributions, saving time, and effort. Even gene tree distributions representing bootstrap replicates might be used as input to *guenomu*, with the caveat that we might not interpret its output in probabilistic terms anymore. Contemplating the uncertainty in the inference of gene trees can provide a better picture of the evolutionary history of species trees.

Here, we have presented *guenomu*, a program capable of accurately estimating the set of likely species trees as well as reducing the uncertainty of sampled gene trees. It is based on a simple model that can be easily expandable, and that incorporates several existing approaches like GTP and the RF supertree. It is also very fast, such that a single run on a data set like the one described by Song et al. (2012) of 447 gene family tree distributions over 37 species would take less than 6 hours using a single processor.

## Supplementary Material

Data available from the Dryad Digital Repository: http://dx.doi.org/10.5061/dryad.74922.

## Funding

This research was financially supported by the European Research Council (grant ERC-2007-Stg 203161-PHYGENOM to D.P.) and Spanish Ministry of Economy and Competitiveness (grant BFU2012-33038 to D.P., FPI fellowship BES-2010-031014 to D.M. at the University of Vigo).

## Appendix

### Pseudocode for the Non-Parametric Generation of Tree Uncertainty

Here are the steps to apply uncertainty to a gene tree T:

1. find maximum branch length $t_{MAX}=MAX_{i}(t_{i})$ over all internal branches i of T, where $t_{i}$ is the branch length;
2. associate to each internal branch i a probability of swap $P_{i}=P_{B}\times (1-t_{i}/t_{MAX})$;
3. with probability $\left(1-P_{T}\right)$, create a copy of T unchanged;
4. Otherwise, with probability $P_{T}$, apply uncertainty as follows. For each internal branch i in postorder (that is, closer to leaves first), using arbitrary root location and assuming left and right children are labelled c1 and c2:
  (a) If c1 is internal but c2 is not, then
    - with probability $P_{c1}/3$ attach the left child of c1 to i;
    - otherwise with probability $P_{c1}/3$ attach the right child of c1 to i;
    - otherwise do nothing;
  (b) If c2 is internal but c1 is not, then
    - with probability $P_{c2}/3$ attach the left child of c2 to i;
    - otherwise with probability $P_{c2}/3$ attach the right child of c1 to i;
    - otherwise do nothing;
  (c) If both c1 and c2 are internal branches, then do one of the following:
    - with probability $P_{c1}(1-P_{c2})$ attach the left or right child of c1 to i, at random;
    - with probability $P_{c2}(1-P_{c1})$ attach the left or right child of c2 to i, at random;
    - with probability $P_{c1}P_{c2}/2$ attach the left or right child of c1 to i, then attach the left or right child of c2 to one of i, the left or right children of c1;
    - with probability $P_{c1}P_{c2}/2$ attach the left or right child of c2 to i, then attach the left or right child of c1 to one of i, the left or right children of c2;
    - with probability $\left(1-P_{c1})(1-P_{c2}\right)$, do nothing;

If the gene tree does not have branch lengths, then a common value is assumed for all branches. Furthermore, if there are several trees in a file, the above procedure is repeated for each one of them, keeping track of their frequencies.
